# Abdominal Aortic Aneurysm: Natural History, Pathophysiology and Translational Perspectives

**DOI:** 10.37825/2239-9747.1037

**Published:** 2022-12-27

**Authors:** Giulio Accarino, Antonio N. Giordano, Martina Falcone, Adriana Celano, Maria G. Vassallo, Giovanni Fornino, Umberto M. Bracale, Carmine Vecchione, Gennaro Galasso

**Affiliations:** a Department of Public Health, Vascular Surgery Unit, University of Naples “Federico II”, I-80126, Naples, Italy; b Department of Medicine, Surgery and Dentistry, University of Salerno, Baronissi, Salerno, Italy; c AOR San Carlo, Cardiovascular Department, Potenza, Italy; d Department of Molecular Medicine and Medical Biotechnologies, University of Naples “Federico II”, I-80126, Naples, Italy; e AOU San Giovanni di Dio e Ruggi D’Aragona, Salerno, Italy

**Keywords:** Abdominal aortic aneurysm, Primary prevention, Atherosclerosis, Smoking, Dyslipidemia

## Abstract

An abdominal aortic aneurysm (AAA) is a degenerative pathology that affects the infrarenal segment of the aorta, leading to its progressive dilatation and eventually rupture. The infrarenal segment is involved in 80% of the aortic aneurisms, and represents alone 30% of all aneurysms.

The natural history of the disease is characterized by the progressive increase of the aortic diameter associated with proportionally higher risk of rupture, particularly for cases with diameter greater than 5.5 cm. In case of rupture the mortality rate is very high, independently from the endovascular or surgical treatment.

The most important risk factors are older age, smoking, hypertension, dyslipidemia, and family history of AAA. The most frequent form is “atherosclerotic”, but infectious, collagen disease-related, immune dysregulation-related, and post-traumatic AAA have also been described. Albeit multiple pathogenetic hypotheses have been proposed, the role of metallo-proteinases in the degeneration of the aortic wall seem to play a central role.

Early detection of AAA is crucial for the identification and treatment before the onset of potentially life-threatening complications. Moreover, the individual risk stratification is fundamental for the clinical management and follow-up.

The growing knowledge about the pathophysiology of AAA has the potential to lead to significant translational advances. The challenge for the next years is to employ bioinformatic and genetic models, also based on artificial intelligence and machine learning approach, to develop novel screening methods and to stratify individuals at higher-risk or in the early stages of AAA.

## 1. Introduction

**Fig. 1:**
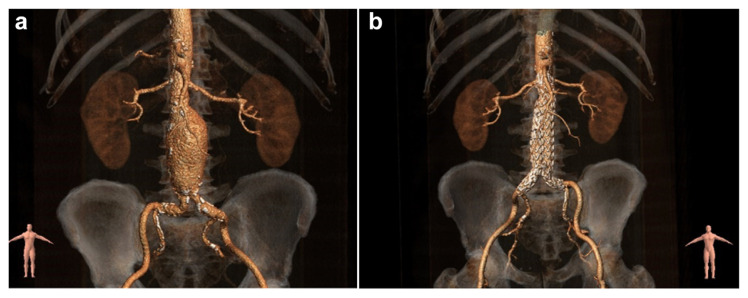
Three-dimensional computed tomography reconstruction of a 70 mm AAA before (panel A) and after (panel B) endovascular treatment.

An abdominal aortic aneurysm (AAA) Fig. 1 Panel A is a focal dilatation or widening of the abdominal aorta related to the weakening of the arterial wall. The infrarenal segment is the most frequently involved, being 80% of the aortic aneurisms, and 30% of all aneurysms [1]. The European Society of Vascular surgery (ESVS) guidelines define aneurysmatic an infrarenal aorta with a diameter greater than 3.0 cm, which is two standard deviations above the mean diameter in men [2], whereas a lower threshold is generally considered for women and patients of Asian ethnicity [3,4] (see Table 1).

A meta-analysis including 26 high-quality observational studies reported an age-related increasing prevalence with age-specific prevalence rate per 100,000 of about 2270 among patients aged 75 to 79 [5].

Although generally asymptomatic, AAA can complicate and become a surgical emergency with a high mortality rate if rupture occurs, which is estimated to arise in about 5% patients/year among subjects with AAA diameter >5.5 cm [6] and become a time-dependent emergency fatal in 100% of non-treated cases [7]. Mortality risk is high also among those admitted in hospital and undergoing prompt open repair, with an estimated 30-day mortality close to 50% [8]. In women, the risk of rupture is also greater than in men for the same aortic diameter, which emphasize the importance of screening in early diagnosis in these subjects [9].

Although multiple genetic and lifestyle risk factors are involved in the development of AAA and the risk of its rupture, how these conditions influence the disease phenotype is not fully understood and is matter of study today.

The aim of this short review was to summarize the pathophysiology, natural history, risk factors underlying AAA, and to provide a translational outlook about their application for screening and clinical management of this high-risk patient population.

## 2. Natural history of AAA

The natural history of AAA is a progressive asymptomatic growth of the aortic diameter driven by a multifactorial degeneration of the aortic wall, which may lead to aortic rupture. Although the majority of AAA are small and associated with low risk of rupture [10], it is crucial to identify patients with AAA and stratify the individual risk of complications and mortality. The main independent predictor of rupture is the aortic diameter, and the follow-up and indication for treatment are currently based on the periodic assessment, as accurate as possible, of this parameter.

In a study conducted between 1995 and 2000, Lederdele et al. found that the risk of rupture was 12%/year at 5.5 cm and markedly increased for diameters greater than 6.5 cm (35%/year) [11]. The mean aneurysm growth rate was 2.2 mm/year and was independent of age and sex. The UKSAT and ADAM trials were designed to investigate variations in life expectancy of patients with small AAAs and confirmed that surgical treatment of AAA <5.5 cm was associated with an increased mortality related to post-surgical and later complications than conservative treatment [12,13].

### 2.1. Differences between abdominal and thoracic aortic aneurism: embryologic and hemodynamic features

Although AAA and thoracic aortic aneurysm (TAA) are commonly considered related pathologies, these conditions are substantially different in terms of epidemiology, pathophysiology and clinical management.

Different aortic segments have different embryogenesis and thus should be considered as separate organs. Ascending aorta, aortic arch and descending thoracic aorta originate from neural crest cells where the media grows by assembling sequential lamellar units maintaining a constant ratio of aortic diameter to medial thickness. The abdominal aorta, conversely, originated at the aortic hiatus from mesoderm cells where the number of lamellar units remains constant while the thickness of each unit expands during maturation [14].

These disparities in aortic cellular origin carries differences in extracellular matrix and microfibril density as well as vascular smooth muscle cell reactivity. This difference in tissue reactivity can also be noted in some metabolic disorders, such as hyperomocysteinemia [15]. When treated with TGF-β, neural crest VSMCs demonstrated increased DNA synthesis and collagen production, while mesodermal VSMCs do not increase collagen output [16].

The infrarenal portion of the aorta is particularly prone to aneurysmatic degeneration, not only for its embryologic origin but also because this segment is subject to blood stream’s impact on the iliac bifurcation and pressure-reflective waves. Increased wall stress and strain caused by the aortic blood stream induces and contributes to the maintenance of an endothelial injury status mediated by sheer stress [17].

## 3. Atherosclerotic AAA

Aneurysm and stenosis are often considered as two different phenotypes of the same disease. In fact, one of the coping mechanisms of a vessel affected by atherosclerotic plaque is the positive remodeling aimed at increasing the diameter of the vessel and maintain blood flow downstream of the stenosis [18].

Although AAA patients show coexistent atherosclerotic disease in one or more vascular districts in 25%–55% of cases, it is not fully understood whether this association is the result of a common underlying mechanism [19].

The presence of peripheral artery disease (PAD) has also been associated with the risk to develop AAA [20], albeit patients with both AAA and PAD have a slower rate of aneurism growth compared with patients with AAA alone, suggesting that PAD might promote the development of AAA but protect against the risk of rapid growth and wall rupture [21].

AAA, coronary artery disease, cerebrovascular disease, or PAD frequently coexist also in the REACH registry [22]. However, some conditions traditionally considered as risk factors for stenotic artery disease, resulted associated with a lower probability of aneurism formation. In particular, diabetes is negatively associated with the risk of AAA and multiple observational studies reported a half prevalence of AAA in diabetic compared to non-diabetic subjects [23–25].

### 3.1. Atherogenic vs immunological hypotheses

AAA is an evolutive disease in which the affected segment is actively remodeled starting from the inside wall, which is coated with intraluminal thrombus (ILT) in about 75% of cases [26]. In the past decades, the AAA was also called “atherosclerotic aneurysm” to describe the main etiology of this condition; however, there were a few studies on lipid metabolism and other underlying mechanisms [27].

Atherosclerosis involves different arteries with different clinical phenotype. The external iliac arteries tend to be aneurysm-resistant, but highly susceptible to atherosclerotic occlusive disease. In male apoE−/− mice infused with Angiotensin II (a common animal model of AAA), atherosclerotic lesions were only detected after development of the aneurysms suggesting that the link between aneurysm development and atherosclerosis may be opposite than previously thought [28]. A balanced view on this subject today is that atherosclerosis and AAA are “processes running in parallel” [29] since in both diseases an atherosclerotic plaque starts forming on an intimal lesion and subsequently replaces the subendothelium [30].

Many authors hypothesized ILT formation as a sort of compensatory mechanism in response to positive arterial remodeling to maintain the vessel lumen [29]. While ILT may be protective against wall stress [31], its permeation by blood or contrast is associated with aneurysm rupture [32]. ILT is a highly active environment since the blood stream constantly replenishes the ILT’s luminal side with fibrinogen and circulating cellular elements, such as platelets, erythrocytes, and immune cells [33]; the ILT entraps these cells, which release oxidative enzymes, proteases and proinflammatory cytokines recruits circulating leukocytes, which then migrate towards the media, and contribute to the arterial wall damage [34].

### 3.2. The role of matrix metalloproteinases

In the highly metabolically active aortic wall, the extracellular matrix disruption and remodeling of the blood vessel are supported by imbalances in the activity of many well-known enzymes, starting with matrix metalloproteinases (MMPs) that have shown to be involved in the pathogenesis of AAA.

The enzymes that are most frequently involved are MMP-1, -2, -3, -9, -12, and –13. The molecular signaling pathways involved in MMP activation include osteopontin, JNK, JAK/stat and AMP-activated protein kinase alpha2. Substrates in the human vasculature for MMP-3, MMP-9, or MMP-14 include collagen, elastin, ECM glycoprotein, and proteoglycans [35].

The association of MMPs with artery wall degeneration began in 1991 when Senior et al., who demonstrated the ability of MMP-3 (stromelysin1) and MMP-9 (gelatinase B) to cleave elastin [36,37].

MMP-9 is the most important MMP in the spectrum of aneurysmal pathogenesis. Genes encoding MMP-9 demonstrates a 12-fold increase in expression within aortic aneurysmal tissue when compared to normal aortic tissue and elevated levels of MMP-9 can also be detected in the serum of patients with an AAA. Other potentially causative MMPs exist within aortic tissue and circulating serum including MMP2, MMP-3, and MMP-12. MMP-2, that may play an important role in aneurysms less than 5 cm. This evidence was reinforced by the lack of elevated MMP-9 levels in the aortic tissue of patients with smaller aneurysms.

While MMP-2 and MMP-9 earned most of the attention in both basic science and clinical research, other MMP have also been implicated in the pathogenesis of AAAs and more research is needed in this field to clarify how each individual enzyme influences aortic remodelling. An example to this intricate network of signaling and coexistence of many different enzymes is that MMP-3 (stromelysin-1) breaks down collagen and other structural proteins of the aortic wall, and also activates other pro-MMPs, which can lead to further activation of active metalloproteinases and continue the breakdown process of the aortic wall [38].

## 4. Risk factors for atherosclerotic AAA

Being an arterial disease, the pathophysiology of AAA has been considered related to the same risk factors of general atherosclerotic occlusive pathology. Although it has been clarified that aneurysmal phenotype shares most of the pathophysiological pathways of stenotic disease, the hazard of the individual risk factors and their interplay is still not fully understood [39].

In this section we report an overview of the main risk factors and their relative weight in the genesis and progression of AAA pathology.

### 4.1. Age and sex

One of the most important risk factors for the development of AAA is age. In a systematic review conducted by Sampson and colleagues in 21 world regions, the annual incidence rate of AAA in 2010 was per 0.84 per 100,000 in 40–44-year-old subjects and 165 in the 75–79 year old subjects, with an increase of almost 200 times between these age groups [5]. The risk of AAA increases with age in both sexes, but in women the development of AAA is generally delayed because of the protective effect of estrogens against hypertension and other AAA-related conditions before menopause [40].

In a prospective, population-based study conducted in Oxfordshire, UK, from 2002 to 2014, acute AAA adverse events were reported in 103 of 92,728 subjects. The incidence/100,000/year was 55 in 65–74-year-old men, 112 in 75–85 years, and 298 in those aged ≥85 years, with 66.0% of overall adverse events occurring in subjects older than 75 years [41].

This evidence shows that the majority of AAA and related acute adverse events occur in subjects over age 75, which may have implications for public health planning and population-based screening strategies.

### 4.2. Smoking

Smoking is the modifiable risk factor most frequently associated with AAA in both men and women, with odds ratios ranging from 3 to 12 [42,43]. The reported relative risk for AAA in current smokers is up to 3 times higher than for coronary artery disease or cerebrovascular disease [44].

In a case–control study including men aged ≥50 years, there was a linear dose response relationship with smoking duration (relative risk of 4% per each year of smoking). The risk of AAA very slowly declined after smoking cessation, suggesting that smoking may play as an initiating event for this condition [45]. In a longitudinal cohort study including 15,792 participants from 1987 to 1989 and followed up through 2013, those who quit smoking during the observation had a 29% lower AAA lifetime risk compared with continuous smokers [46].

In animal models exposed to tobacco smoke after a relatively minor aortic elastase injury there was an increase in elastin degradation and aneurysm size, independent of matrix metalloproteinase expression [47].

Smoking has been demonstrated to increase the plasmatic level of nitro-tyrosine in both smokers and patients with chronic obstructive pulmonary disease, and fibrinogen is among the plasma proteins that are nitrated enhancing its immunogenicity. The aortic aneurysm antigenic protein-40 kDa (AAAP-40), which have been purified from the adventitia of the human aorta, has seven loci for potential tyrosine nitration and, noteworthy, have homologies to fibrinogen beta (FB-b) [48]. Many authors hypothesized that smoking may induce immunoreactive reaction against the aortic wall based on the homologies between AAAP-40 and FB-b (Hirose H.a, 1998). Indeed, anti-fibrinogen antibodies promote AAA in mouse models and anti-fibrinogen antibodies are detectable in humans with AAA [49].

### 4.3. Hypertension

Luminal blood pressure is the most obvious contributor to the arterial wall stress and hypertension has been suggested as a risk factor for AAA, but several cross-sectional and case–control studies are not entirely consistent with a statistically significant positive association between blood pressure and AAA [50–52].

In a meta-analysis including 18 studies focused on small AAA, defined for diameters between 3.0 and 5.4 cm, there was a markedly increased risk of rupture with growing mean arterial pressure, suggestive for the importance of antihypertensive medications in individuals with a small AAA, with targets of 130/80 mmHg [9]. Noteworthy, they found no significant association between blood pressure value and AAA growth.

A more recent meta-analysis of 21 cohort studies, conversely, showed a 66% higher risk of developing AAA in patients with hypertension compared to those without [53]. The authors also reported a non-linear association between diastolic blood pressure and the development of AAA, emphasizing diastolic blood pressure having a greater impact than systolic blood pressure on the risk of AAA.

The association between AAA growth and hypertension has been investigated in experimental models showing both elastic lamellar disruption and inflammatory cell infiltration within the wall of the aorta of hypertensive rats, along with a positive correlation between AAA growth rates and controlled blood pressure values [54].

Hypertension seems also to accelerate the progression of AAA through an increase in matrix degradation and inflammation mediated by matrix metalloproteinase (MMP)-2, –3, –9, –12 and intercellular adhesion molecules, induced by the upregulation of NFκB gene [55].

### 4.4. Dyslipidemia

High-density lipoprotein cholesterol (HDL-C) levels have been associated with lower probability of being affected by AAA [56], independently by treatment with lipid-modifying medications. This association may be also related to the anti-inflammatory and antioxidant effects of HDL-C that might be protective against AAA [57].

Conversely, low-density lipoprotein cholesterol (LDL-C) serum levels seem to increase the risk of AAA [58], albeit the quality and the quantity of low-density-lipoproteins (LDL) seem to exert a direct influence. In fact, small dense LDL has demonstrated to have the strongest association with the presence of AAA [59].

However, there is insufficient evidence on how high levels of total cholesterol or different cholesterol particles contribute to the development of AAA. It needs to be noted that most of the observational studies include AAA people but not a comparator group from the general population [60].

Despite the association with the presence of AAA, dyslipidemia seems not to influence the AAA growth or the risk of rupture [61].

### 4.5. Family history and genetics

Approximately 6–20% of patients with AAA have a positive family history and the relative risk of first-degree relatives of persons diagnosed with AAA was approximately doubled compared to those without a family history. There is robust epidemiologic evidence that heritability contributes to increased risk of aneurysm formation [62]. This risk has been estimated to be 71 times higher in monozygotic twins if one of them has been diagnosed with AAA, emphasizing the importance of genetics in the pathophysiology of AAA. Neither the gender of the index person nor of the first-degree relative influenced the higher risk of AAA patients with positive family history [63]. Despite this robust epidemiological evidence, AAA has not been clearly associated yet with polymorphisms or mutations in non-syndromic genes. Three genome-wide studies investigating approximately 1000 single nucleotide polymorphisms in 100 candidate genes have found only weak associations [64] except for rare hereditary diseases such as Marfan Syndrome or Ehlers-Danlos Syndrome [65]. As an example, one of the most studied genes for AAA formation is the antioncogene DAB2IP has a probability of P 1/4 4.6 10, but the risk odds ratio is only 1.21 [66].

## 5. Specific aetiologies of AAA

### 5.1. Mycotic AAA

A mycotic aneurysm is caused by bacterial, fungal, or viral infection. Its risk is higher in immune-compromised patients including subjects with acquired immunodeficiency syndrome, treated with high-dose glucocorticoids, chemotherapy, malignancy, etc. The incidence is low since it represents 1–3% of AAA.

The most common pathogens involved in mycotic AAA are *Staphylococcus aureus* (28–71%), and *Salmonella* (15%). A pre-existing intimal injury is thought to be a precondition to enable the infectious intima colonization, resulting from bacterial seeding in a susceptible vessel area. A mitotic aneurysm can also be initiated from a iatrogenic bacterial inoculation caused by puncture or surgery.

The mixture of arterial injury and bacterial seeding results in the colonization of the intima stimulating the release of pro-inflammatory cytokines, with neutrophils chemotaxis and consequent MMP activation [67].

A particular form of aortitis is caused by *Treponema pallidum* that obliterates the vasa vasorum and induces necrosis of the elastic fibers and connective tissue in the aortic media [68]. Aortitis is reported in 70–80% of syphilitic patients left untreated after the primary infection, and 10% of patients may be complicated by aortic aneurysm, aortic regurgitation, and coronary ostia stenosis [69]. Indeed, the transverse scars caused by *T. pallidum* are suspected to tight up the aortic layers and protect against the risk of dissection.

Albeit in the past century tertiary syphilis was the most common cause of AAA, this is currently a very rare condition due to the widespread availability of diagnostic tests and antibiotics [70].

### 5.2. Inflammatory aneurysm

Inflammatory AAA represents a relatively small subgroup of AAA, ranging from 2% to 18% of all AAA and have only been described recently thanks to higher quality imaging and tissue testing. Their identification is fundamental due to the distinct pathophysiology, diagnosis, clinical management, and treatment strategy.

Inflammatory AAA is distinguished from atherosclerotic form for the thickened aneurysm wall often associated with a fibrosis that involves nearby structures such as the duodenum, ureter, and inferior vena cava [71]. Patients tend to be younger than those with purely atherosclerotic AAA probably because this specific etiology is related to autoimmune or inflammatory diseases that become clinically relevant earlier [72]. Inflammatory markers, such as white blood cell count, erythrocyte sedimentation rate, C reactive protein are generally found elevated and it is common to find serum positivity for anti-nuclear antibody and elevation of IgG4 typical of autoimmune response [73,74]. The aneurysm and the thickened aortic wall with periaortic inflammation and fibrosis have a typical CT sign, the so-called “mantle sign” and, if surgically treated, the appearance of white, sparkling, perianeurysmal fibrosis is specific of inflammatory aortic aneurysm [75].

## 6. Translational perspectives

### 6.1. Screening for AAA

AAA is typically asymptomatic until life-threatening complications such as rupture occurs, and routine screening should be considered a public health measure in order to reduce AAA-related mortality. Since AAA is estimated to affect almost 8% of general population over 65 years, an effective screening health policy should be widespread available both in hospital and outpatient settings, swiftly executed, and safe.

Screening has acquired a very important role in the reduction of the mortality from all vascular diseases thanks to increasing awareness of vascular pathology and the joint venture between peripheral and third-level centers according to a “hub and spoke” model. Many authors also reported screening campaigns being associated with a significant reduction of AAA emergency treatment and with an increase in elective surgery [76,77].

The setting up a screening protocol start with the identification of the population group with the highest probability of benefit. In AAA, this means including patients aged at least 65, smokers, and with family history of aneurysmal disease, hypertension, and ectasia in other arterial districts (e.g. popliteal artery). The simplest and most cost-effective approach in outpatient setting is represented by the physical examination of the abdomen aimed at evaluating the presence of a pulsating dilation with uneven margins in the epi-mesogastric region. A more sensitive approach, albeit less feasible in peripheral centers by non-trained operators, would be the abdominal ultrasound examination. Although abdominal ultrasound examination is not a novel technique, it requires equipment and experienced personnel. A large Swedish study [78] conducted over a period of 8 years and enrolling over 300,000 male subjects (over 84% of the subjects target population) undergoing ultrasound examination reported an average mortality reduction from aortic diseases of about 4%/year. However, it is still debated if the estimated reduction of mortality may justify the cost of extensive ultrasound screening campaigns.

Beyond clinical and ultrasound assessments, the prospective of rapid molecular testing (e.g. point of care) to identify the early pathophysiological processes underlying the development of the disease is intriguing for the scientific community. Although attractive, this is an arduous and still distant goal, since the role of genetic material and gene regulation in the development and evolution of the AAA is a very dynamic field of study.

A study conducted on surgical samples of arterial wall form patients with acute aortic dissections, showed that ITM2C, an integral membrane protein, was under-expressed while miR-107-5p complex was over-expressed [79].

Studies of non-coding RNAs. from Zhang F and colleagues [80,81] have described the role played by non-coding RNAs, such as long non-coding RNA (lncRNA), circular RNA (circRNA) and microRNA (miRNA), in AAA development. The circRNAs, as the competing endogenous RNA, act as a competitive endogenous RNA and compete with the miRNA through the response element to the miRNA itself. The miRNA molecules, in turn, down-regulate the mRNA expression of protein-coding genes by binding to complementary sequences initiating a feedback induced regulation of protein synthesis [82,83]. These mechanisms of dysregulation and altered interaction between circRNA-miRNA-mRNA may be the biological basis of the genesis and development of AAA.

In an experimental study by Han Y and colleagues [84], post-transcriptional modifications play a relevant role in the functional regulation of circRNA specifically involved in aneurysmal degeneration of abdominal aortic wall.

Novel translational insights are provided by Wang et al. [79], who suggest that non-coding RNAs can hypothetically be used as biomarkers for screening and patients’ prognostic stratification.

Thanks to the use of the GEO (Gene Expression Omnibus) database, at least six different dysregulations on the circ-RNA-miRNA-mRNA gene network present in the aorta affected by dilated pathology were found by Zhang H [83]. Furthermore, it was possible for the first time to reconstruct the circRNA-miRNA-mRNA network through the GEO database albeit further research is needed to evaluate its significance in AAA pathogenesis.

The challenge for the coming years is to use bioinformatic and genetic models to reconstruct current knowledge, also by using machine learning approach, and develop screening techniques based on AAA pathophysiology in order to identify higher risk subjects and anticipate the disease onset in a early phase.

### 6.2. Management of the AAA

Most scientific societies agree to surgically treat an AAA that presents a diameter of at least 55 mm or even less in presence of symptoms, specific anatomic features, or increased risk of rupture determined by the coexistence of specific conditions (e.g. rapid growth, absence of thrombus).

While risk factor control may be useful to reduce AAA incidence and smoking cessation may help slightly reduce growth rate, no pharmacological therapy has proved to be effective in slowing or stopping AAA growth, and avoid rupture, despite wide range of drug classes has been investigated [85].

Some anti-hypertensive drugs such as beta-blockers have been proposed in this patients’ setting, but randomized studies have not demonstrated a beneficial effect of propranolol on AAA growth. Similar findings were reported for angiotensin- converting enzyme inhibitors and other medication such as doxycyclin and azithromycin [86,87]. Statins have also been investigated for their potential to promote AAA stabilization, but study results were inconsistent and not conclusive [88,89]. However, statin therapy is recommended in these patients owing to the association with higher long-term survival irrespective of the effect on AAA [90]. Metformin treatment in diabetics seems has also been associated with lower AAA growth [91], but further studies are needed to confirm this preliminary finding.

Best clinical practice, as advised by the most recent guidelines, the most recent of which was published by the Italian Society of Vascular and Endovascular Surgery in 2022 [92], involve carefully evaluating every patient to estimate aneurysm rupture risk versus surgical risk and life expectancy before offering surgical or endovascular repair, which remains the only curative solution Fig. 1 Panel B [93,94]. After careful evaluation the patient with a small aneurysm (<5.5 cm) will be offered either a close surveillance or, based on age and anatomy, an early treatment [95,96]. Endovascular aneurysm repair may be considered for most of the since currently available devices allow to candidate subjects with different aortic anatomic characteristics. The life expectancy of the individual patient influences the choice between traditional open surgery and endovascular treatment since endovascular treatment is associated with better 1-year survival is linked to a substantially higher 5-year reintervention rate [97]. In patients with long life expectancy (relatively young and with few comorbidities), open surgery may be an the best option since it is associated with an acceptable perioperative risk and with better long-term aortic related complication rate than endovascular treatment [98].

## 7. Conclusions

AAA is still one of the leading cause of death worldwide albeit many advances has been made in unraveling its underlying multifactorial pathogenesis, clinical management and treatment, in recent years. As surgery remains the standard of care in big AAA, it is associated with a substantial rate of complications, particularly in the emergency setting.

The early detection of AAA and the identification of the individual profile associated with the highest probability to develop AAA represent the objective of translational research in the coming years. Although surgery will probably remain the recommended treatment for big AAA, the understanding of the pathogenetic mechanisms may lead to the development of pharmacological strategies for treatment of patients with small AAA.

All these advances will enable not only to reduce the morbidity and mortality of AAA, but also to reduce the number of surgical interventions and costs to the health care system.

## Figures and Tables

**Table 1:** Main enzymes involved in AAA pathogenesis and their role.

Metalloproteinases	Etiopathogenetic role	Reference
MMP-9 (gelatinase B)	Cleaving elastine	Ramella M. et al. Am J Transl Res. 2017 PMID: 29312500
MMP-2 (gelatinase A)	Cleaving collagene I, II, III, etiopathogenetic role in aneurysms less than 5 cm in diameter	Maguire E.M., Pharmaceuticals (Basel), 2019 doi: 10.3390/ph12030118
MMP-12 (metalloelastase)	Cleaving elastine	Gona K., J Med Chem, 2020 doi: 10.1021/acs.jmedchem.0c01514
MMP-3 (stromelysin-1)	Cleaving elastine and activating other pro-MMPs.	Rabkin S.W., Prog Mol Biol Transl Sci, 2017 doi: 10.1016/j.matbio.2007.07.001
